# Supplementary material for: Identification of photoperiod-induced specific miRNAs in the adrenal glands of Sunite sheep (Ovis aries)
Source: Front Vet Sci. 2022 Jul 22;9:888207. doi: 10.3389/fvets.2022.888207 (PMC9354845; doi:10.3389/fvets.2022.888207)
Supplement: Supplementary file 1 [file Data_Sheet_1.ZIP › Supplementary file/miRDeep2 file.zip!/miRDeep2 file/Know_Structure/LP42A1/oar-mir-487b.pdf]

miRBase precursor : oar-mir-487b
Total read count : 2521
oar-miR-487b-5p read count : 35
oar-miR-487b-3p read count : 2481
remaining reads : 5

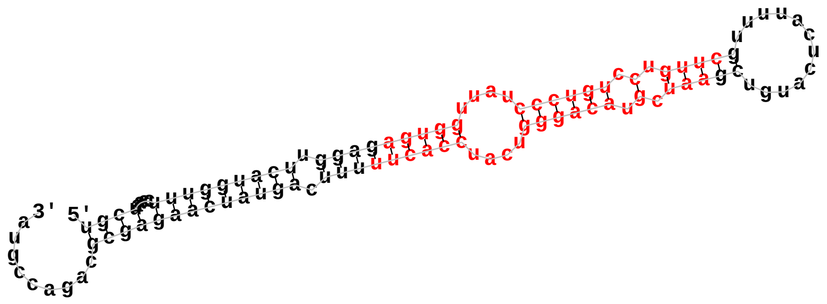

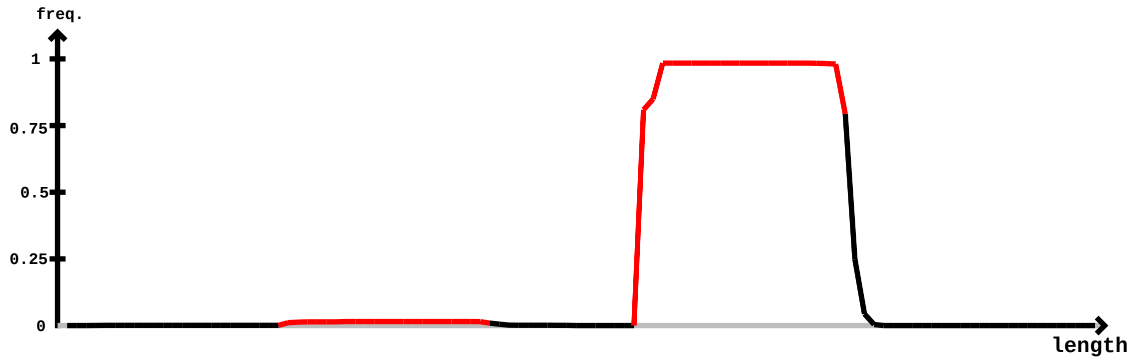


```
                                                                   oar-miR-487b-3p
                          oar-miR-487b-5p
5'- ugcuaaccuuugguacuuggagagugguuaucccuguccuguucguuuuacucaugucgaaucguacagggucauccacuuuuucaguaucaagagcgcagaccgua  -3'    exp
    .(((....(((((((((.((((((((((....((((((.(.(((((...........))))).).))))))....)))))))))).)))))))))))).........   reads   mm   sample
    ..cuaaccuuugguacuuggaga....................................................................................   1       0    seq
    ...uaaccuuugguacuuggaga....................................................................................   1       0    seq
    ......................agugguuaucccuguccugu.................................................................   1       0    seq
    ......................agugguuaucccuguccuguu................................................................   13      0    seq
    ......................agugguuaucccuguccuguuc...............................................................   9       0    seq
    ......................agugguuaucccuguccuguucg..............................................................   2       0    seq
    .......................gugguuaucccuguccuguu................................................................   1       0    seq
    .......................gugguuaucccuguccuguucg..............................................................   5       0    seq
    .......................gugguuaucccuguccuguucgu.............................................................   1       0    seq
    ........................ugguuaucccuguccuguuc...............................................................   1       0    seq
    ........................ugguuaucccuguccuguucg..............................................................   2       0    seq
    ............................uaucccuguccuguucguuuua.........................................................   1       0    seq
    ............................uaucccuguccuguucguuuuacu.......................................................   2       0    seq
    ...........................................................aaucguacagggucaucc..............................   2       0    seq
    ...........................................................aaucguacagggucaucca.............................   2       0    seq
    ...........................................................aaucguacagggucauccac............................   5       0    seq
    ...........................................................aaucguacagggucauccacu...........................   460     0    seq
    ...........................................................aaucguacagggucauccacuu..........................   1326    0    seq
    ...........................................................aaucguacagggucauccacuuu.........................   244     0    seq
    ...........................................................aaucguacagggucauccacuuuu........................   1       0    seq
    ............................................................aucguacagggucauccacu...........................   8       0    seq
    ............................................................aucguacagggucauccacuu..........................   35      0    seq
    ............................................................aucguacagggucauccacuuu.........................   48      0    seq
    ............................................................aucguacagggucauccacuuuu........................   10      0    seq
    ............................................................aucguacagggucauccacuuuuu.......................   1       0    seq
    .............................................................ucguacagggucauccacu...........................   4       0    seq
    .............................................................ucguacagggucauccacuu..........................   11      0    seq
    .............................................................ucguacagggucauccacuuu.........................   226     0    seq
    .............................................................ucguacagggucauccacuuuu........................   89      0    seq
    .............................................................ucguacagggucauccacuuuuu.......................   9       0    seq
```
